# Microwave Curing of FA- and MK-Based Geopolymer Gels: Effects on Pore Structure, Mechanical Strength, and Heavy Metal Leachability

**DOI:** 10.3390/gels11070507

**Published:** 2025-06-30

**Authors:** Yanhui Dong, Runhui Gao, Yefan Li, Fuchen Wang

**Affiliations:** 1 Key Laboratory of Deep Petroleum Intelligent Exploration and Development, Institute of Geology and Geophysics, Chinese Academy of Sciences, Beijing 100029, China; 2 College of Earth and Planetary Science, University of Chinese Academy of Sciences, Beijing 100049, China; 3 MOE Key Laboratory of Groundwater Circulation and Evolution, China University of Geosciences, Beijing 100083, China; gaorunhui@126.com; 4 School of Energy and Mining Engineering, China University of Mining and Technology, Beijing 100083, China; lyf2495076637@163.com (Y.L.); w_fuchen@163.com (F.W.)

**Keywords:** microwave curing, geopolymer gels, compressive strength, heavy metal immobilization, fly ash and metakaolin-based materials

## Abstract

Microwave curing has proven to be a highly effective method for enhancing the structural integrity, compressive strength, and heavy metal immobilization performance of geopolymer (GP) gels. For fly ash-based GP gels, optimal compressive strength (126.84 MPa) and minimal heavy metal ion leaching (0.01 mg/L) were achieved under microwave irradiation at 100 W for 75 s. Similarly, metakaolin-based GP gels reached peak compressive strength (76.84 MPa) and reduced heavy metal leaching (0.44 mg/L) under 440 W irradiation for 60 s. Microwave energy significantly accelerates geopolymerization by promoting the aggregation of dispersed particles, rapidly forming a dense, block-like matrix. This accelerated densification enhances the mechanical properties of GP gels within minutes. Moreover, the dense matrix structure effectively encapsulates heavy metal ions, minimizing their leaching through a combination of physical encapsulation and chemical bonding. In summary, microwave treatment significantly enhances both mechanical performance and heavy metal immobilization, offering a practical pathway for sustainable applications.

## 1. Introduction

The global climate crisis necessitates a transition to sustainable construction materials. With concrete production accounting for over 7% of global CO_2_ emissions, innovative solutions are required to mitigate its environmental impact while maintaining durability [1]. GP-based materials offer a low-carbon alternative with enhanced long-term stability [2]. Microwave curing accelerates geopolymerization while minimizing energy consumption, aligning with climate imperatives and resource efficiency goals. GP gels are amorphous inorganic polymeric binding materials based on aluminosilicates, synthesized through a polymerization process [3,4]. GP gels exhibit remarkable mechanical properties and durability, making them a promising alternative to conventional cement-based materials. Studies have demonstrated their superior compressive strength, freeze–thaw resistance, sulfate corrosion resistance, and chloride penetration resistance, which are crucial for long-term structural stability [5,6]. Their outstanding mechanical properties and durability have garnered significant attention in both research and the construction industry [7]. As alternatives to Portland cement composites, GP gels have been employed in specialized applications such as refractory coatings, fiber-reinforced composites, and waste immobilization [8]. Formed through chemical reactions between alkaline solutions and raw materials rich in silicon and aluminum, GP gels exhibit unique advantages, including low permeability, long-term stability, and exceptional resistance to acidic media [9]. These features address the limitations of traditional cement-based solidification techniques in treating heavy metal contamination [10]. Microwave curing offers a rapid, uniform heating method with low energy consumption, penetrating materials volumetrically for efficient large-scale geopolymerization [11]. Studies confirm its potential in ultra-high-performance concrete (UHPC) fabrication [12], yet further research is needed to optimize exposure and power levels to prevent degradation and enhance industrial applicability.

In the field of heavy metal pollution remediation, GP gels have emerged as a research hotspot due to their distinct advantages. Their three-dimensional network structure, composed of silicon–oxygen and aluminum–oxygen tetrahedra, provides a robust foundation for heavy metal immobilization. The immobilization mechanisms primarily include physical encapsulation, ion exchange, and adsorption. Physical encapsulation embeds heavy metals or their oxides within the gel structure [11,12,13]. Ion exchange enables heavy metal ions to replace cations or anions within the GP structure [14]. Adsorption leverages the porous structure of GP gels to trap heavy metals [15,16].

Several factors influence the effectiveness of heavy metal immobilization by GP gels, including the silicon-to-aluminum (Si/Al) ratio, heavy metal dosage, ion valence state and form, reducing agents, leaching methods, reaction temperature, and curing/adsorption time. The Si/Al ratio directly affects structural stability, with an optimal range enhancing immobilization efficiency [17]. Excessive heavy metal content may weaken mechanical strength and increase leaching concentrations [18]. For instance, reducing Cr^6+^ to Cr^3+^ significantly improves the immobilization efficiency of chromium [19]. GP-based immobilization effectively reduces the mobility of heavy metals, mitigating their threats to ecosystems and human health. Additionally, synthesizing GP gels using raw materials like metakaolin and fly ash not only promotes waste utilization but also provides a sustainable solution for heavy metal pollution management, aligning with environmental protection principles and sustainable development goals.

However, the efficiency of geopolymerization can be significantly slowed down without an external heat source. Conventional thermal curing relies on heat transfer from the material’s surface to its core through convection, conduction, and radiation, driven by a thermal gradient [20]. Conventional thermal curing of GP materials typically requires temperatures ranging from 40 °C to 90 °C and durations from 24 to 72 h, depending on the precursor type and activator concentration [4,21,22]. These prolonged curing conditions limit the scalability and energy efficiency of GP production, especially in industrial applications. Recently, microwave technology has emerged as a promising alternative, offering rapid and uniform heating, short processing cycles, and high energy penetration [23]. Unlike traditional methods, microwave heating efficiently transfers heat without direct contact with the GP, resulting in a clean, non-polluting process that does not generate secondary waste [20]. These advantages make microwave technology a highly promising approach to enhancing GP performance. Traditional curing methods often require high temperatures (up to 100 °C) and extended durations, which are impractical and uneconomical for industrial applications [24].

Microwave heating converts electrical energy into an electromagnetic field, which is then transformed into thermal energy [25,26,27]. This volumetric heating process heats particles of all sizes simultaneously, minimizing thermal stress and producing a more refined internal material structure [28,29]. Under microwave curing, the depolymerization–polycondensation reaction reduces the activation energy of the powder and accelerates the reaction. Compared to traditional curing times (spanning tens of hours), microwave curing achieves equivalent mechanical performance within minutes [30,31,32]. This process also accelerates moisture removal and promotes the gel’s polycondensation, enabling the rapid reconstruction of the silica-alumina framework. Ultimately, a robust three-dimensional silica-alumina network is formed, significantly enhancing the curing speed and overall performance of the GP [30,33]. Previous studies have confirmed that microwave curing significantly accelerates geopolymer setting and early strength development. For example, Aschoff et al. found that metakaolin-based geopolymers achieved comparable strength within minutes under microwave radiation, compared to several days under ambient conditions [34]. Similarly, Watanabe and Kobayashi reported that optimized microwave curing enhances matrix densification and reduces porosity [35]. These findings highlight the efficiency and potential of microwave curing for geopolymer systems. While microwave radiation offers a novel approach to curing GP gels by leveraging rapid, volumetric heating, challenges remain in optimizing exposure times and power levels to prevent material degradation. Further studies are needed to fully understand its effects on pore structure, mechanical performance, and heavy metal immobilization capabilities.

This study investigates high-performance GP gels subjected to microwave radiation, analyzing changes in their micro-pore structure and curing performance after treatment. It examines the impact of microwave power settings and duration on GP performance, focusing on mechanical strength and heavy metal immobilization efficiency. By exploring different microwave curing conditions, this research aims to leverage the advantages of microwave radiation to accelerate the GP curing process, reduce the total curing cycle, and enhance both mechanical strength and environmental remediation capabilities. Finally, this study evaluates the relationship between pore structure and mechanical properties under varying microwave times and power levels.

## 2. Results and Discussion

### 2.1. Morphology from SEM

**Figure 1:**
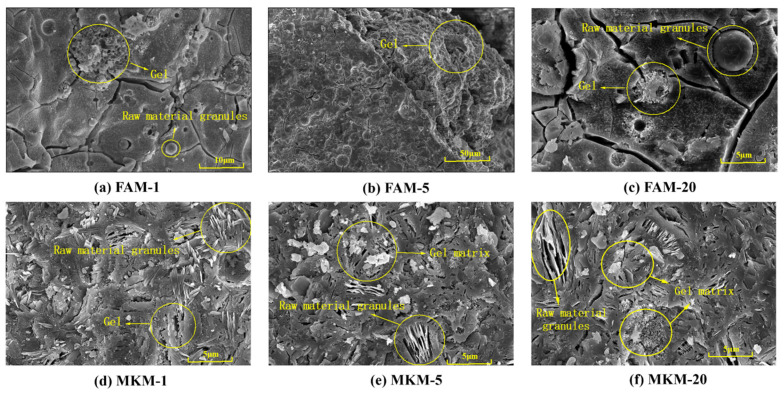
SEM images of microwave-curing GP gels.

Figure 1 displays the SEM images of GP gels cured under different microwave powers, where Figure 1a–c depicts the FA-based GP gels and Figure 1d–f represents the MK-based GP gels. Comparing Figure 1a,b with Figure 1d,e reveals that microwave radiation duration significantly affects the microstructure of the GP gels. Figure 1b shows the emergence of abundant hydration gels on the surface, while Figure 1e shows larger and more concentrated matrix blocks, suggesting that extended heating promotes geopolymerization reactions, forming denser gels. Figure 1c,f, showing GP gels subjected to high microwave power for extended durations, reveals damaged gel structures and the presence of larger cracks. This is attributed to the rapid water evaporation and excessive moisture loss caused by the high microwave energy input, which inhibits raw material dissolution and gel formation. Additionally, newly formed hydration phases (e.g., N-A-S-H) lose hydrogen due to dehydration, and high temperatures induce significant internal stress, leading to structural disruption and crack formation.

Microwave-cured GP gels form block-like matrices on their surfaces, indicating that microwave curing accelerates the geopolymerization process, achieving a matrix comparable to that of 28 days of conventional curing within a much shorter time frame. This highlights the higher efficiency of microwave curing over conventional curing.

### 2.2. XRD and FT-IR Analysis

**Figure 2:**
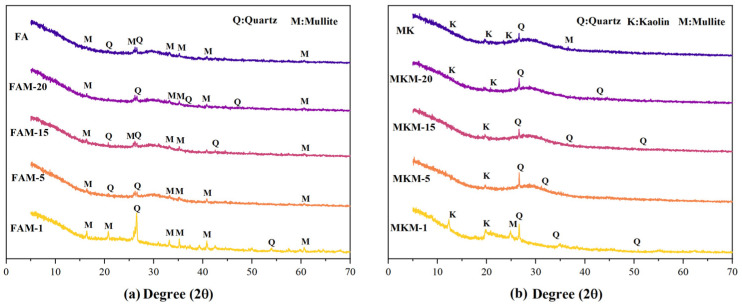
X-ray diffractometry patterns of different GP gels: (**a**) FA-based GP; (**b**) MK-based GP. FAM(MKM): FA represents fly ash, MK represents metakaolin.

The XRD diffraction patterns of fly ash-based (Figure 2a) and metakaolin-based (Figure 2b) GP gels reveal their phase compositions. For fly ash-based GP gels, the raw material FA showed quartz and mullite as the primary phases, distinct from metakaolin. After microwave curing, diffraction peak shapes and intensities in the 2θ range of 20°–30° changed, with significant transformations observed in FAM-15 and FAM-20. Peaks corresponding to the quartz phase near 2θ ≈ 26° disappeared or weakened. For metakaolin-based GP gels, raw material MK primarily comprises quartz, mullite, and kaolin phases. After microwave curing at varying powers and durations, changes in quartz diffraction peak intensity and position near 2θ ≈ 26° were observed. For instance, in low-power MKM-1, the quartz peak was stronger, whereas, in high-power MKM-15 and MKM-20, the peak weakened, and new diffraction peaks appeared near 2θ ≈ 45°, indicating phase transformation and new phase generation.

The dissolution of MK and FA in alkaline activators facilitated the decomposition of internal minerals, forming [AlO4]^−^ and [SiO4], which underwent condensation reactions. Despite differences in raw material phases, observations under various microwave powers and durations revealed distinct trends in the number and intensity of quartz peaks.

**Figure 3:**
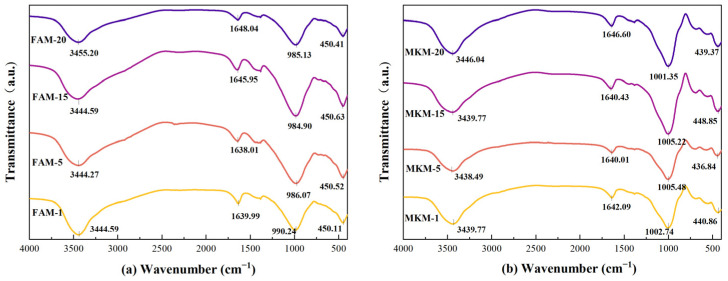
FT-IR spectra of different GP gels: (**a**) FA-based GP; (**b**) MK-based GP.

FTIR spectra of fly ash-based (Figure 3a) and metakaolin-based (Figure 3b) GP gels further elucidate structural differences. Broad bands near 3440 cm^−1^ and weak peaks around 1640 cm^−1^ correspond to O-H stretching vibrations of interlayer water molecules and H-O-H bending vibrations, indicating the presence of structural water. Peaks near 1640 cm^−1^ are attributed to trapped water molecules within aluminosilicate structures. Metakaolin-based GP gels showed characteristic peaks around 1000 cm^−1^, while fly ash-based GP gels exhibited peaks near 986 cm^−1^, corresponding to asymmetric stretching vibrations of Si-O-Si and Si-O-Al bonds. For instance, the peak at 1005.48 cm^−1^ in MKM-5 shifted to 1001.35 cm^−1^ in MKM-20 (Δ = 4.13 cm^−1^), while the peak at 986.07 cm^−1^ in FAM-5 shifted to 984.90 cm^−1^ in FAM-20 (Δ = 1.17 cm^−1^). These peaks shifted with variations in microwave power and duration, with lower wavenumber shifts correlating to increased aluminum atom proportions in the structure, indicative of changes in Si-O-T bond vibrations.

Si-O-T (T: Si or Al) intensity signifies a more disordered structure (condensation), associated with greater high-wavenumber shifts, implying maximum substitution of Si by Al. This highlights the critical role of optimal microwave curing conditions in GP reactions. Differences in absorption peak shifts between metakaolin- and fly ash-based GP gels may stem from variations in their chemical compositions and structures, influencing the chemical reactions and structural transformations occurring during microwave curing.

### 2.3. Pore Characteristics from NMR

#### 2.3.1. Pore Characteristics Under Different Microwave Powers

**Figure 4:**
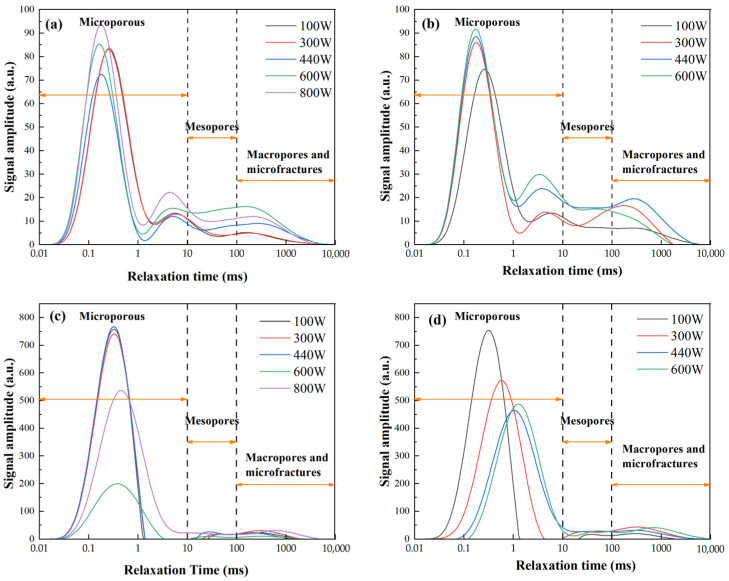
NMR *8* spectra of microwave-cured GP at different powers: (**a**) FA-30 s, (**b**) FA-90 s, (**c**) MK-30 s, (**d**) MK-90 s.

The *T*_2_ relaxation spectra of GP gels subjected to selected microwave powers are shown in Figure 4. These spectra intuitively reflect the micro-pore distribution of GP gels under various microwave heating conditions and reveal the connectivity nature of their pore systems.

Across all heating durations, the *T*_2_ spectra of FA-based GP gels exhibit a characteristic “one primary and two secondary” three-peak pattern, as shown in Figure 4a,b. The primary peak, located within the *T*_2_ range of 0.01~1 ms, is prominent and distinct. In contrast, the secondary peaks appear within the *T*_2_ range of 1~10 ms, with decreasing intensity, and the third peaks appear around 100 ms. This three-peak distribution highlights the structural features of the internal pore system. First, the significant height of the primary peak indicates that micropores dominate the GP’s pore system. Second, the smooth transitions between the primary and secondary/third peaks suggest good connectivity between micropores and meso-/macropores, forming an interconnected pore network rather than isolated pores. The relaxation time ranges correspond to distinct pore structures: 0.01–10 ms (micropores), 10–100 ms (mesopores), and 100–10,000 ms (macropores and microfractures).

The *T*_2_ relaxation spectra reflect pore structure characteristics in GP gels. The area under the curve indicates total pore volume, while the curve shape reveals pore size distribution-higher *T*_2_ values correspond to larger pores. A pronounced first peak confirms that micropores constitute a major portion of the total pore volume, indicating their dominant structural role.

Under low-power microwave curing, the first peak is lower and sharper. As microwave power increases (100–440 W), the peak shifts leftward and decreases in area, signifying micropore reduction. In contrast, secondary peaks (>10 ms) increase in area and height, indicating mesopore growth. This suggests that moderate microwave energy promotes geopolymerization and restricts micropore expansion, enhancing densification. Excessive power, however, induces rapid water evaporation, enlarging pores and reducing structural integrity.

For MK-based GPs, a similar trend is observed. At low power, a single micropore-dominant peak appears. With increased power, the curve shifts rightward (larger pores) and peak height diminishes (fewer pores), reflecting pore coarsening. Microwave-induced volumetric heating enhances water mobility, raw material dissolution, and network formation, yielding a denser structure.

At higher powers, micropore and mesopore peaks begin to merge, indicating pore interconnection. Rapid steam generation damages fine pores, which evolve into larger voids and eventually macropores or microcracks.

In summary, *T*_2_ spectra confirm that moderate microwave power promotes matrix densification, while excessive energy leads to pore expansion and degradation. These findings are critical for optimizing microwave curing to enhance GP microstructure and performance.

#### 2.3.2. NMR T_2_ Characteristics Under Different Microwave Durations

**Figure 5:**
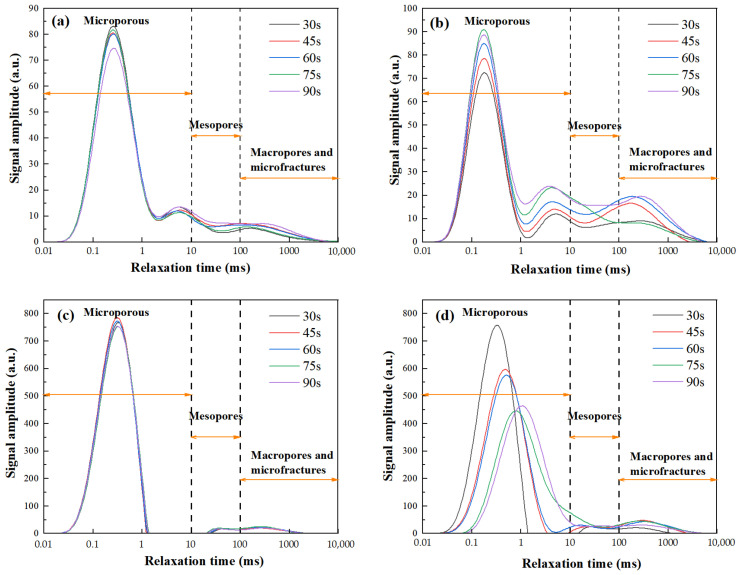
NMR *T*_2_ spectra of microwave-cured GP at different times: (**a**) FA-100 W, (**b**) FA-440 W, (**c**) MK-100 W, (**d**) MK-440 W.

Figure 5 illustrates the NMR *T*_2_ spectra of FA-based and MK-based GP gels subjected to varying durations of microwave radiation. For FA-based GP gels, i.e., those shown in Figure 5a,b, for longer microwave radiation durations, the *T*_2_ spectra of GP gels reveal minimal shifts in the peak position of the micropore region compared to those treated for shorter durations. However, the area of the mesopore peak increases, indicating a rise in the number of mesopores and an expansion of the overall pore system. In samples exposed to shorter microwave durations, the amplitude of the micropore peak is relatively low.

This is attributed to the fact that short-duration, low-energy microwave radiation accelerates geopolymerization, promoting the formation of gels and densifying the microstructure. In contrast, for longer microwave durations, the amplitude of the peaks increases, suggesting that the accumulated internal heat and increased radiation energy significantly activate the GP’s microwave-absorbing components. This heightened activity results in structural damage and pore expansion.

In MK-based gels, prolonged microwave radiation shifts the *T*_2_ peaks rightward, denoting increased pore size and reduced pore quantity. As exposure extends from 30 s to 90 s, micropore peak amplitude declines, while mesopore amplitude rises markedly. This suggests a transformation from micropores to mesopores, driven by microwave-induced thermal expansion. Enhanced interconnection between pore types is also observed, increasing total porosity and internal structural complexity.

In summary, extended microwave duration reduces micropores, increases mesopores, and improves pore connectivity in GP gels. These changes, driven by internal thermal expansion, highlight the importance of controlling radiation time to optimize microstructure and performance.

#### 2.3.3. Porosity Evolution Under Different Microwave Durations and Powers

**Figure 6:**
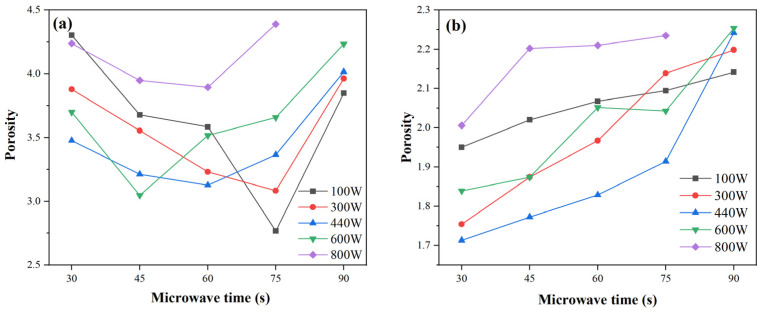
Porosity of GP gels under different microwave powers and durations. (**a**) Fly ash-based GP gels at different microwave durations and powers; (**b**) metakaolin-based GP gels at different microwave durations and powers.

Figure 6 presents the porosity of FA- and MK-based GP gels under various microwave curing times and power levels, derived from NMR data in Section 2.3.

For FA-based gels (Figure 6a), porosity initially decreases and then increases with longer exposure times, reaching a minimum between 45 and 75 s. Under short exposure durations (30–60 s), porosity decreases and then rises with power, while longer exposures (>75 s) lead to a continuous increase. This trend reflects two competing effects: moderate microwave energy accelerates geopolymerization and micropore development, reducing porosity; excessive power or duration, however, causes rapid water loss and thermal stress, leading to pore expansion, microcracks, and increased porosity.

In MK-based gels (Figure 6b), porosity shows a similar pattern, decreasing initially with increased power and reaching a minimum at 440 W, but rising steadily under prolonged exposure (90 s). This suggests that initial conventional curing may more effectively initiate geopolymerization in MK systems. At short durations (30 s), MK gels achieve their lowest porosity, indicating rapid densification. However, as microwave energy increases, evaporation effects dominate, reversing the trend.

These porosity changes align with compressive strength trends discussed in Section 2.4. At low microwave energy, rapid volumetric heating enhances pozzolanic activation and material densification, improving strength. Peak performance was observed at 100 W, 75 s for FA-based gels, and 440 W, 30 s for MK-based gels. Beyond optimal conditions, excessive heating causes vapor escape, pore coarsening, and microcracking, leading to strength decline. Additionally, non-uniform dielectric properties result in localized overheating and internal stresses, further degrading structural integrity.

### 2.4. Compressive Strengths

#### 2.4.1. Mechanical Properties Under Different Microwave Power and Durations

**Figure 7:**
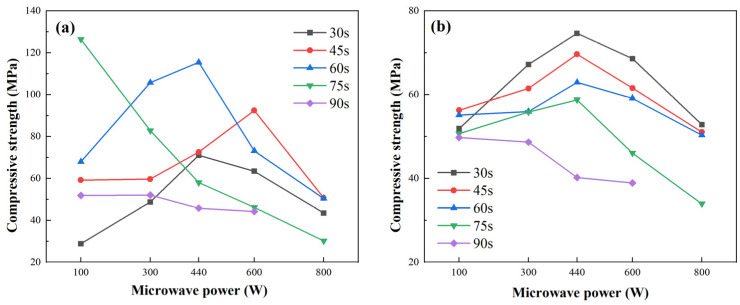
Curing performance of GP gels under different microwave power levels and radiation durations. (**a**) Fly ash-based GP gels under different microwave power levels. (**b**) Metakaolin-based GP gels under different microwave power levels.

Figure 7 illustrates that the compressive strength of both fly ash- and metakaolin-based geopolymer gels increases initially and then declines with rising microwave power or exposure time. For fly ash-based gels, strength ranged from 30 to 126.84 MPa, peaking at 100 W for 75 s. Metakaolin-based gels showed smaller variation, with a maximum of 76.84 MPa at 440 W for 60 s.

This non-linear trend reflects the dual effects of microwave curing: moderate power enhances geopolymerization by accelerating heating and pozzolanic activation, while excessive energy input (e.g., >300 W or >45 s) induces structural degradation. Peak strength occurred at 100 W/75 s (fly ash) and 440 W/30 s (metakaolin). All strength values represent the mean of three replicates.

At moderate energy inputs, microwave radiation accelerates heating, enhancing pozzolanic activation and promoting geopolymerization, thereby improving matrix densification and mechanical strength. However, excessive power or extended duration causes rapid water loss, pore expansion, microcracking, and structural degradation. High energy input also induces non-uniform temperature fields due to the differing dielectric properties of components, resulting in internal stresses and potential thermal runaway.

Prolonged curing further destabilizes the polymer network, weakens chemical bonds, and reduces heavy metal immobilization efficiency. Insufficient exposure fails to initiate gel formation, while overexposure leads to matrix dehydration and shrinkage, causing both surface and internal cracking. Additionally, alkali leaching under prolonged radiation may further compromise strength.

Overall, compressive strength development under microwave curing follows a non-linear trend, improving under optimal conditions and declining with excess energy input due to increased porosity, thermal stress, and microstructural damage. Reported strength values represent averages from three replicate specimens.

#### 2.4.2. Correlation Between Porosity Characteristics and Mechanical Strength

**Figure 8:**
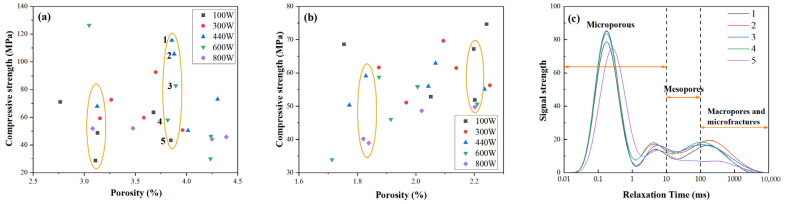
Strength vs. porosity plots of microwave-conditioned GP gels: (**a**) FA-based GP; (**b**) MK-based GP.1-5:(75 s-100 W/300 W 440 W/600 W/800 W); (**c**) pore size distribution of GP gels with a porosity of about 3.8%. The numbers 1–5 represent five samples at 100 W, 300 W, 440 W, 600 W, and 800 W under 75 s.

Figure 8a shows the relationship between porosity and compressive strength for microwave-cured FA-based GP gels. The data do not exhibit a consistent linear trend. For instance, some GP gels with the same porosity level of approximately 3.8% (e.g., samples 1–5) demonstrate substantial variations in compressive strength, ranging from 40 MPa to 110 MPa. A similar trend is observed in MK-based GP samples, as depicted in Figure 8b. Despite having the same porosity, compressive strength varies significantly.

When analyzed in conjunction with the pore size distribution in Figure 8c, a pattern emerges: at the same porosity level, samples with higher compressive strength tend to exhibit reduced peak heights in the pore size distribution curve. This suggests that pore size, rather than overall porosity, plays a crucial role in determining compressive strength. Specifically, as compressive strength increases, the proportion of micropores decreases, resulting in a lower peak in the pore size distribution.

Overall, as microwave power increases, compressive strength decreases. While the pore size distribution curves do not shift laterally, the total pore volume increases, indicating a reduction in micropore volume. The curve areas corresponding to mesopores, macropores, and microcracks, however, remain largely unchanged. For example, in curve 5, the pore size distribution shifts slightly to the right, indicating an increase in micropore size. Although the peak height decreases, the total porosity remains relatively unchanged. This expansion of micropores contributes to the observed reduction in compressive strength.

These findings suggest that the development of micropores is a critical factor influencing the compressive strength of GP gels. GP gels with smaller and more uniformly distributed micropores exhibit higher compressive strength, while those with larger micropores or poorly connected pore structures tend to have reduced strength. Thus, controlling micropore development is key to optimizing the mechanical performance of GP gels.

### 2.5. Heavy Metal Leachability

**Figure 9:**
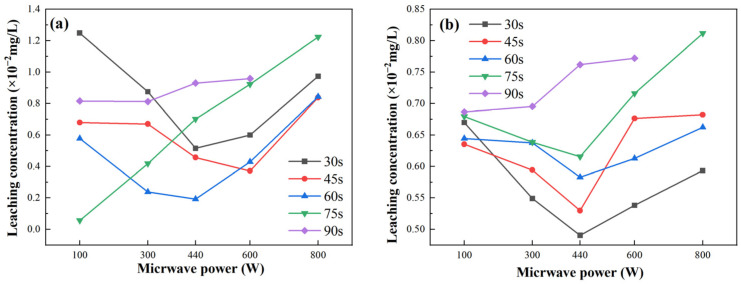
Leaching performance of GP gels under different microwave power levels and radiation durations. (**a**) Fly ash-based GP gels under different microwave power levels; (**b**) metakaolin-based GP gels under different microwave power levels.

Figure 9 illustrates the leaching performance of fly ash-based and metakaolin-based GP gels under varying microwave power levels and radiation durations. For fly ash-based GP gels, as shown in Figure 9a, the leaching rate initially decreases and then increases with increasing microwave power. The overall leaching performance increases with a microwave radiation duration of 75 s. The World Health Organization (WHO) set the maximum allowable concentration of total chromium in drinking water at 0.05 mg/L. The highest leaching rate for fly ash-based GP gels, approximately 1.3 × 10^−2^ mg/L, occurs at a microwave power of 100 W. For metakaolin-based GP gels, Figure 9b shows that the leaching rate varies with microwave power, reaching a peak value of 0.82 × 10^−2^ mg/L at a microwave power of 800 W. Overall, the highest leaching rates for both fly ash-based and metakaolin-based GP gels occur at a microwave power of 800 W and a radiation time of 90 s.

In general, the ion leaching concentration is inversely proportional to the compressive strength of the GP. Higher compressive strength corresponds to a more stable three-dimensional network structure, which facilitates the encapsulation and immobilization of heavy metal ions. Moreover, the formation of more aluminum–oxygen tetrahedral network structures enhances the binding of heavy metal ions through chemical bonding, embedding them securely within the GP framework. Additionally, the chemical bonds formed in the structure are more stable under these conditions.

The leaching rates of heavy metal ions show an inverse trend with compressive strength. For both fly ash-based and metakaolin-based GP gels, microwave radiation significantly accelerates the hydration reaction, thereby enhancing compressive strength. Additionally, microwave treatment promotes the binding of Cr^3+^ ions with C-S-H-Cr and C-S-H gels, effectively reducing the leaching of chromium ions.

The observed reduction in leaching rates under microwave treatment highlights its ability to improve the encapsulation and immobilization of heavy metals within GP gels, providing a robust and efficient solution for heavy metal stabilization.

### 2.6. Relationship Between Mechanical Strength and Leaching Performance

**Figure 10:**
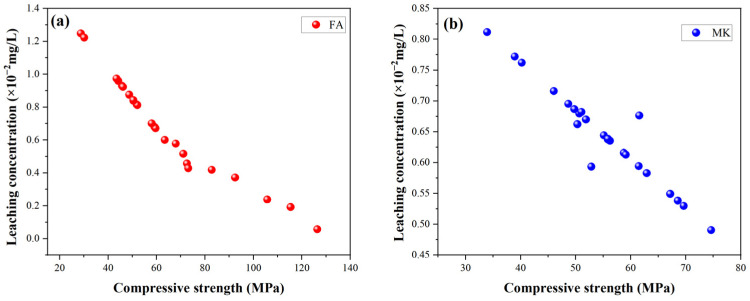
Correlation between compressive strength and leaching concentration of GP gels. (**a**) FA-based GP gels; (**b**) MK-based GP gels.

Figure 10 illustrates the relationship between compressive strength and leaching concentrations for FA-based and MK-based GP gels. Under the same microwave power and exposure time, a negative correlation is evident between compressive strength and leaching performance. However, both GP gels exhibit similar trends as microwave power and time increase. During the microwave treatment process, when power and time are within an optimal range, the internal structure of the GP is likely to gradually form and strengthen. This structural formation and reinforcement may simultaneously improve compressive strength and reduce leaching rates.

Appropriate microwave radiation facilitates the involvement of more raw material particles in hydration, leading to higher nanocrystal content in microwave-cured GP gels. Comparing SEM micrographs of microwave-cured and oven-cured GP gels, it is observed that microwave-cured specimens exhibit more voids but with smaller pore sizes. These pores primarily arise from water evaporation during the curing process. Water, as a polar molecule, absorbs microwave energy efficiently, rapidly heating to its boiling point and escaping from the sample, resulting in smaller pore sizes and increased porosity. These microstructural changes contribute to the development of compressive strength and reduce their leachability.

For example, Figure 1e shows larger and more concentrated matrix blocks, suggesting that extended heating promotes geopolymerization reactions, forming denser gels. This not only enhances the compressive strength of the GP but also encapsulates heavy metal ions within the matrix, reducing their leachability.

Generally, the leaching concentration of ions is inversely proportional to the compressive strength of the solidified matrix. Higher compressive strength corresponds to a more stable three-dimensional network structure, which effectively encapsulates and immobilizes heavy metal ions. Additionally, the formation of more aluminum–oxygen tetrahedral frameworks enhances the chemical bonding of heavy metal ions within the GP structure, embedding them securely into the matrix. The associated chemical bonds are also more stable, further reducing the likelihood of ion leaching.

However, as microwave power increases beyond the optimal range, the high microwave energy induces significant internal stress, compromising the stability of the network structure and the chemical bonds within the GP. This destabilization can lead to the release of previously bonded heavy metal ions, reverting them to a free state. Consequently, this degradation in structural integrity adversely affects both the leaching performance and solidification efficiency of the GP.

These findings highlight the need for careful optimization of microwave power and exposure time to achieve a balance between compressive strength and leaching resistance, ensuring the effective solidification of heavy metals while maintaining the mechanical performance of GP gels.

## 3. Conclusions

Microwave curing has been demonstrated to significantly enhance the structural integrity, compressive strength, and curing performance of GP gels. For fly ash-based GP gels, optimal compressive strength of 126.84 MPa and minimal heavy metal ion leaching of 0.01 mg/L were achieved under 100 W microwave irradiation for 75 s. Similarly, metakaolin-based GP gels exhibited their highest compressive strength of 76.84 MPa and heavy metal ion leaching of 0.44 mg/L under 440 W microwave irradiation for 60 s. The rapid and uniform heating provided by microwave energy facilitates the aggregation of dispersed, incompletely reacted GP particles into larger-scale, block-like matrix structures. This process accelerates the densification of the GP gel, significantly enhancing compressive strength within a short timeframe. Furthermore, the dense matrix structure formed during microwave curing effectively immobilizes heavy metal ions by encapsulating them within the GP network and binding them chemically, thereby minimizing their leaching.

The integration of microwave technology into GP synthesis represents a transformative step in advancing sustainable construction materials. It not only accelerates production cycles but also improves material performance, offering a viable pathway for developing stronger, more durable, and environmentally friendly building materials. However, challenges remain in scaling microwave curing processes to industrial applications, particularly in the design and optimization of large-scale microwave reactors. Future studies should explore strategies to adapt microwave curing for large-scale concrete elements and onsite construction processes, ensuring uniform energy distribution and curing efficiency in practical scenarios. Additionally, optimizing microwave reactor configurations to accommodate varying material compositions and thicknesses will be crucial for industrial implementation.

Additionally, further research is required to evaluate the long-term durability and environmental resilience of microwave-cured GP gels, ensuring their effectiveness and stability under various service conditions. Investigating its economic feasibility, energy efficiency, and compatibility with existing construction workflows will further strengthen its potential as a scalable curing solution. These advancements hold significant potential for creating innovative materials that meet the growing demands of sustainable construction and environmental remediation.

## 4. Materials and Methods

### 4.1. Materials

The fly ash (FA) used in this study was purchased from Gongyi Refractories Co., Ltd. (Zhengzhou, China), with a particle size of 18 μm. Its calcium content is less than 10%, classifying it as low-calcium fly ash. Metakaolin (MK), a high-activity aluminosilicate mineral formed by the high-temperature calcination of kaolinite, was sourced from Chenyi Refractory & Abrasives Co., Ltd. (Zhengzhou, China), with a particle size of 3.75 μm. The chemical composition of FA and MK is shown in Table 1. The activator used for the geopolymerization process was water glass, i.e., sodium silicate solution, a commonly employed activator. It was purchased from Porun Foundry Materials Co., Ltd. (Henan, China), as industrial-grade water glass with an initial modulus of 2.23. Analytical-grade NaOH (≥96.0% purity), obtained from Shanghai Aladdin Biochemical Technology Co., Ltd. (Shanghai, China), was used to adjust the modulus of the water glass. The sodium silicate was modified during the experiments to adjust the modulus of the alkaline activator to 1.4. This study primarily focused on immobilizing heavy metals. Chromium (Cr), specifically in the trivalent state (Cr(III)), was selected as a representative heavy metal due to its common presence, broad application, lower toxicity compared to other heavy metals, and ease of immobilization. Chromium nitrate (Cr(NO_3_)_3_·9H_2_O), an analytical-grade compound, was purchased from Shanghai Aladdin Biochemical Technology Co., Ltd. (Shanghai, China), and used as the source of Cr(III) in the experiments.

### 4.2. Synthesis of GP Gels

FA and MK powders were first weighed and thoroughly mixed as the base materials. Following this, the alkaline activator solution was added to the mixture in accordance with the specified L/S of 1.6 for MK and 0.7 for FA. The mixture was then transferred to a cement paste mixer. Initially, the mixture was stirred at low speed to ensure uniform mixing of all components, a process that lasted for 10 min. After mixing, any paste adhering to the blades and container walls was scraped back into the center of the container. The thoroughly mixed slurry was poured into polytetrafluoroethylene molds of two dimensions: 40 × 40 × 40 mm^3^ for mechanical strength testing and Φ25 × 20 mm^3^ for heavy metal leaching analysis. The filled molds were then subjected to vibration compaction to expel internal air bubbles. The molds containing the slurry were placed in a 40 °C constant-temperature oven for 4 h for thermal curing. Then, microwave radiation power settings of 100 W, 300 W, 440 W, 600 W, and 800 W were used for the samples, with heating times ranging from 30 s to 90 s in 15 s intervals (Table 2). After curing, the samples were demolded. The resulting GP samples were placed into a microwave oven for radiation treatment at specific power levels and durations. Following microwave treatment, the GP gels were allowed to cool naturally to room temperature before undergoing characterization and testing.

### 4.3. Characterization and Testing of GP Gels

Fourier Transform Infrared (FTIR) spectroscopy (Bruker VERTEX 70 V, Billerica, MA, USA) was used to analyze the chemical structure of the samples, capturing infrared spectra in the 400–4000 cm^−1^ range to identify chemical bonds and functional groups. Mineralogical phases were examined via X-ray diffraction (XRD) (PANalytical X’Pert PRO, Almelo, The Netherlands) with a 2θ range of 5–70° at a scan speed of 0.01°/s. Structural analysis was conducted using MDI Jade 6.5 software and compared with the Crystallography Open Database. Scanning Electron Microscopy (FEI Nova Nano SEM450, Hillsboro, OR, USA) provided detailed visualization of pore structures and microstructural features.

Mechanical properties were assessed using a Universal Hydraulic Servo Tester (E64.305 300 kN, MTS, Eden Prairie, MN, USA). Heavy metal leaching was evaluated through inductively coupled plasma mass spectrometry (ICP-MS, PerkinElmer SCIEX ELAN DRC-e, Waltham, MA, USA) on GP gels encapsulating 1 mg/mL Chromium (III). Solidified samples were crushed to ≤3 mm particles, mixed with deionized water (1:10 solid-liquid ratio), sealed, and oscillated at 25 °C (110 oscillations/min) for 8 h, followed by 16 h of standing. The supernatant was filtered through a 0.45 μm membrane before ICP-MS analysis.

The pore structure of the samples was characterized using Nuclear Magnetic Resonance (NMR) analysis, in which the GP solid material to be tested was saturated and filled with water. In order to fully describe the transverse relaxation time *T*_2_ of a fluid in a porous medium, it can be expressed with the help of a mathematical model:

(1) $$\frac{1}{T_{2}}=\frac{1}{T_{2B}}+\frac{1}{T_{2S}}+\frac{1}{T_{2D}}=\frac{1}{T_{2B}}+\rho \frac{S}{V}+\frac{{D_{m}\left(GT_{E}\gamma \right)}^{2}}{12}$$

where *T*_2B_ represents free relaxation time, *T*_2S_ is surface relaxation time, and *T*_2D_ corresponds to diffusion relaxation time. *ρ* denotes transverse surface relaxation strength, *S* is pore surface area, and *V* refers to pore volume. *D*_m_ indicates the molecular diffusion coefficient, *G* represents the magnetic field gradient, *T*_E_ is the echo time, and *γ* is the magnetic spin ratio.

The transverse relaxation time (*T*_2_) of the GP samples was determined using the Carr–Purcell–Meiboom–Gill (CPMG) pulse sequence. The measurement parameters included a 2000 ms waiting time, 0.2 ms echo time, 8000 echoes, and four sampling repetitions, ensuring precise characterization of the material’s relaxation behavior. Analysis of the results indicates that surface relaxation ($\rho \frac{S}{V}$) is the dominant factor in T_2_, primarily due to the extensive pore surface area in geopolymer gels, which enhances adsorption-desorption interactions. In contrast, free relaxation (*T*_2B_) and diffusion relaxation (*T*_2D_) play relatively minor roles.

## Figures and Tables

**Table 1:** Chemical composition of aluminosilicate solid binders and the utilized water glass.

(wt.%)	SiO_2_	Al_2_O_3_	Fe_2_O_3_	Cl	CaO	SO_3_	H_2_O	OH^−^	Na_2_O	Else
Fly ash	46.6	38.2	3.2	0.015	4.2	2.1	0.85	1.2	/	3.635
Metakaolin	53.00	41.50	0.80	/	/	/	/	/	/	4.7
Water glass	31.00	/	/	/	/	/	57.45	/	11.55	/

**Table 2:** Experimental design of microwave curing for FA- and MK-based GP gels.

ID	Raw Materials	Microwave Power (W)	Microwave Time (s)	ID	Raw Materials	Microwave Power (W)	Microwave Time (s)
FAM-1	MK 3.75 μm	100	30	MKM-1	FA 18 μm	100	30
FAM-2			45	MKM-2			45
FAM-3			60	MKM-3			60
FAM-4			75	MKM-4			75
FAM-5			90	MKM-5			90
FAM-6		300	30	MKM-6		300	30
FAM-7			45	MKM-7			45
FAM-8			60	MKM-8			60
FAM-9			75	MKM-9			75
FAM-10			90	MKM-10			90
FAM-11		440	30	MKM-11		440	30
FAM-12			45	MKM-12			45
FAM-13			60	MKM-13			60
FAM-14			75	MKM-14			75
FAM-15			90	MKM-15			90
FAM-16		600	30	MKM-16		600	30
FAM-17			45	MKM-17			45
FAM-18			60	MKM-18			60
FAM-19			75	MKM-19			75
FAM-20			90	MKM-20			90
FAM-21		800	30	MKM-21		800	30
FAM-22			45	MKM-22			45
FAM-23			60	MKM-23			60
FAM-24			75	MKM-24			75

## Data Availability

The original contributions presented in this study are included in the article. Further inquiries can be directed to the corresponding author.

## References

[1] Xiao J., Zou S., Poon C.S., Sham M.L., Li Z., Shah S.P. (2025). We Use 30 Billion Tonnes of Concrete Each Year—Here’s How to Make It Sustainable. Nature.

[2] Akter S.T., Hawas A. (2025). Current Insight on Eco-Friendly Concrete: A Review. Buildings.

[3] Matsimbe J., Dinka M., Olukanni D., Musonda I. (2022). Geopolymer: A Systematic Review of Methodologies. Materials.

[4] Li Y., Dong Y., El-Naggar M.R., Wang F., Zhao Y. (2024). The Influence of Particle Size and Calcium Content on Performance Characteristics of Metakaolin- and Fly-Ash-Based Geopolymer Gels. Gels.

[5] Li C., Sun H., Li L. (2010). A review: The comparison between alkali-activated slag (Si + Ca) and metakaolin (Si + Al) cements. Cem. Concr. Res..

[6] Nodehi M., Taghvaee V.M. (2022). Alkali-Activated Materials and Geopolymer: A Review of Common Precursors and Activators Addressing Circular Economy. Circ. Econ. Sustain..

[7] Askarian M., Tao Z., Adam G., Samali B. (2018). Mechanical properties of ambient cured one—Part hybrid OPC—Geopolymer concrete. Constr. Build. Mater..

[8] Dave N., Sahu V., Misra A.K. (2020). Development of geopolymer cement concrete for highway infrastructure applications. J. Eng. Des. Technol..

[9] Guo X., Zhang L., Huang J., Shi H. (2017). Detoxification and solidification of heavy metal of chromium using fly ash—Based geopolymer with chemical agents. Constr. Build. Mater..

[10] Huang X., Huang T., Li S., Muhammad F., Xu G., Zhao Z., Yu L., Yan Y., Li D., Jiao B. (2016). Immobilization of chromite ore processing residue with alkali—Activated blast furnace slag—Based geopolymer. Ceram. Int..

[11] Li X., Wang J., Li S., Gao X., Shi Z. (2025). Application of Low-Temperature Microwave Radiation on the Preparation of UHPC. Low-carbon Mater. Green Constr..

[12] Xu S., Xu W., Chen Y., Li J., Li Y. (2024). Enhancement of Microwave Heating Technology for Emulsified Asphalt Mixtures Using SiC-Fe₃O₄ Composite Material. Materials.

[13] Xu J.Z., Zhou Y.L., Tang R.X. (2006). Study on the solidification of heavy metals by fly ash based geopolymers. J. Build. Mater..

[14] El-Eswed B.I., Yousef R.I., Alshaaer M., Hamadneh I., Al-Gharabli S.I., Khalili F. (2015). Stabilization/solidification of heavy metals in kaolin/zeolite based geopolymers. Int. J. Miner. Process..

[15] Cheng T.W., Lee M.L., Ko M.S., Ueng T.H., Yang S.F. (2012). The heavy metal adsorption characteristics on metakaolin—Based geopolymer. Appl. Clay Sci..

[16] Yu Z., Song W., Li J., Li Q. (2020). Improved simultaneous adsorption of Cu(II) and Cr(VI) of organic modified metakaolin—Based geopolymer. Arab. J. Chem..

[17] Fansuri H., Anisatun I.M., Fatmawati A., Utomo W.P. (2016). Cd and Cr Cation Immobilization by Using Geopolymer Based on PT. IPMOMI Fly Ash. Mater. Sci. Forum..

[18] Wang Y., Han F., Mu J. (2018). Solidification/stabilization mechanism of Pb(Ⅱ), Cd(Ⅱ), Mn(Ⅱ) and Cr(Ⅲ) in flyash based geopolymers. Constr. Build. Mater..

[19] Chen J., Wang Y., Zhou S., Lei X. (2017). Reduction/immobilization processes of hexavalent chromium using metakaolin—Based geopolymer. J. Environ. Chem. Eng..

[20] Graytee A., Sanjayan J.G., Nazari A. (2018). Development of a high strength fly ash-based geopolymer in short time by using microwave curing. Ceram. Int..

[21] Patil A.A., Chore H.S., Dode P.A. (2014). Effect of curing condition on strength of geopolymer concrete. Adv. Concr. Constr..

[22] Lopes A., Lopes S., Pinto I. (2023). Influence of Curing Temperature on the Strength of a Metakaolin-Based Geopolymer. Materials.

[23] Sata V., Sathonsaowaphak A., Chindaprasirt P. (2012). Resistance of lignite bottom ash geopolymer mortar to sulfate and sulfuric acid attack. Cem. Concr. Compos..

[24] Hong S., Kim H. (2019). Effects of Microwave Energy on Fast Compressive Strength Development of Coal Bottom Ash-Based Geopolymers. Sci. Rep..

[25] Luo J., Hunyar C., Feher L., Link G., Thumm M., Pozzo P. (2004). Theory and experiments of electromagnetic loss mechanism for microwave heating of powdered metals. Appl. Phys. Lett..

[26] Datta A.K., Rakesh V. (2013). Principles of Microwave Combination Heating. Compr. Rev. Food Sci. Food Saf..

[27] Kim B.J., Yi C., Kang K.I. (2015). Microwave Curing of Alkali-Activated Binder Using Hwangtoh without Calcination. Constr. Build. Mater..

[28] Shearer C.R., Foudazi A., Hashemi A., Donnell K.M. Microwave characterization of fly ash geopolymerization. Proceedings of the 2016 IEEE International Instrumentation and Measurement Technology Conference Proceedings.

[29] Hong S., Kim H. (2019). Robust synthesis of coal bottom ash-based geopolymers using additional microwave heating and curing for high compressive strength properties. Korean J. Chem. Eng..

[30] Samantasinhar S., Singh S. (2020). Effects of Curing Environment on Strength and Microstructure of Alkali-Activated Fly Ash-Slag Binder. Constr. Build. Mater..

[31] Leung C.K.Y., Pheeraphan T. (1997). Determination of Optimal Process for Microwave Curing of Concrete. Cem. Concr. Res..

[32] Bakarev T. (2005). Geopolymeric Materials Prepared Using Class F Fly Ash and Elevated Temperature Curing. Cem. Concr. Res..

[33] Zhang X., Yang Y., Ong C.K. (1997). Study of Early Hydration of OPC-HAC Blends by Microwave and Calorimetry Technique. Cem. Concr. Res..

[34] Aschoff J., Partschefeld S., Schneider J., Osburg A. (2024). Effect of Microwaves on the Rapid Curing of Metakaolin- and Aluminum Orthophosphate-Based Geopolymers. Materials..

[35] Watanabe Y., Kobayashi T. (2023). Geopolymers Prepared by Microwave Treatments. Advanced Ceramics.

